# Environmental Asthma Reduction Potential Estimates for Selected Mitigation Actions in Finland Using a Life Table Approach

**DOI:** 10.3390/ijerph120606506

**Published:** 2015-06-09

**Authors:** Isabell Katharina Rumrich, Otto Hänninen

**Affiliations:** Department of Health Protection, National Institute for Health and Welfare (THL), Kuopio 70210 Finland; E-Mail: Otto.Hänninen@thl.fi

**Keywords:** asthma, risk factor, protective factor, tobacco, particulate matter, dampness, pets, Burden of Disease, prevalence

## Abstract

Aims: To quantify the reduction potential of asthma in Finland achievable by adjusting exposures to selected environmental factors. Methods: A life table model for the Finnish population for 1986–2040 was developed and Years Lived with Disability caused by asthma and attributable to the following selected exposures were estimated: tobacco smoke (smoking and second hand tobacco smoke), ambient fine particles, indoor dampness and mould, and pets. *Results:* At baseline (2011) about 25% of the total asthma burden was attributable to the selected exposures. Banning tobacco was the most efficient mitigation action, leading to 6% reduction of the asthma burden. A 50% reduction in exposure to dampness and mould as well as a doubling in exposure to pets lead each to a 2% reduction. Ban of urban small scale wood combustion, chosen as a mitigation action to reduce exposure to fine particles, leads to a reduction of less than 1% of the total asthma burden. Combination of the most efficient mitigation actions reduces the total asthma burden by 10%. A more feasible combination of mitigation actions leads to 6% reduction of the asthma burden. *Conclusions:* The adjustment of environmental exposures can reduce the asthma burden in Finland by up to 10%.

## 1. Introduction

Asthma is a chronic inflammatory disease of the respiratory system, which is characterized by swelling and narrowing of the bronchial tubes leading to wheezing, chest tightness, breathlessness, and coughing [1]. The prevalence of asthma has increased since the 1960s in the Scandinavian countries, including Finland [2]. In 2007 the prevalence of self-reported doctor-diagnosed asthma in adults (20–69 years) was as high as 9.4% in Helsinki [3]. The severity and controllability of symptoms seems dependent on patient age, with older asthmatics spending more days in hospital due to their asthma on average compared to young asthmatics [4]. The diagnosis of asthma is based on clinical features such as the demonstration of reversible expiratory airflow obstruction, because the pathogenesis is not understood well enough to identify a special test or biomarker specifically for asthma [5].

The Finnish Ministry of Social Affairs and Health set up a National Asthma Programme in 1994–2004 aiming at improving asthma care and to limit the expected increase in costs due to asthma. By improving medication, education and self-management, it was possible to reduce the hospital days due to asthma from 32,000 to 15,000 in 2000 to 2010 [4], as well as the costs per asthmatic patient from €1611 to €1031 in 1993–2003 [6]. 

Asthma is a multi-causal disease with exposure to environmental, lifestyle, as well as prenatal and genetic factors being identified as risk and protective factors [7]. In available epidemiological studies these factors are either associated with asthma onset (incidence) or the occurrence or worsening of asthma symptoms (prevalence). However, a scientific justification for the selection of either of it is not given [8,9,10,11,12,13,14]. To our knowledge there are no studies so far aiming at the quantification of the asthma attributable burden of disease and the corresponding identified risk and protective factors in Finland, in order to identify the fraction of asthma that could be prevented by nationwide changes in exposure to risk and protection factors. 

This work aims at the quantification of the fraction of asthma that is attributable to chosen exposures in order to define suitable mitigation actions. The selected mitigation actions are tested and their reduction potential is compared to identify the best mitigation actions combined into two mitigation scenarios. 

## 2. Material and Methods

A general description of the model, the used input data and applied trend estimations is presented here. More details for the interested readers on the model and the underlying literature review are reported in the corresponding author’s thesis [15].

### 2.1. Asthma Statistics

In order to characterize asthma in Finland from 1986 to 2012 and to forecast the future from 2013 to 2040, a life table model was developed [15]. Age-specific asthma prevalence data were obtained from the Finnish Social Security Institution (KELA) as the total number of entitlements to reimburse asthma medication per calendar year and year of patient age from 1986 to 2012. These entitlements are based on a doctor diagnosis of asthma including lung function tests and further reviewed by KELA. Patient age specific future trends until 2040 were estimated using exponential best fits to the observed data from 2008 to 2012 [15]. KELA is funded by taxes and every Finnish citizens and individual permanently registered in Finland is a KELA member and has with that the right for medical expenses reimbursement. Medication for prevention or good health cannot be reimbursed. Additionally, the most economic package size has to be purchased and pharmacists must suggest the cheapest drug (generic), but the doctor or patient can decline it. The price that can be reimbursed is regulated via a Reference pricing list and the reimbursement is mostly deducted in the pharmacy by presenting the KELA card. If the medication is more expensive than on the reference pricing list, the patient has to pay the difference [16].

The life table does not account for immigration and emigration. The impact of these was tested by comparing observations and model prediction for the population size change in 1986–2012, where the error was between 0.01% (1986) and 0.3% (2012) overestimation of the life table compared to the observed numbers.

### 2.2. Identification of Risk and Protective Factors

Risk and protective factors were identified by a systematic literature review [15]. The inclusion in the model was based expert judgement of the plausibility of causality, the magnitude of the attributable fraction of asthma (practical significance) and the feasibility of adjusting the exposures in the whole population. Consequently, selected risk factors were exposure to: (1) tobacco (active smoking and second hand smoke (SHS)), (2) fine particles (PM_2.5_), (3) dampness and mould in buildings, and (4) daily exposure to pets (cat and dog). Exposure to pets was assumed to pose a risk in the atopic sub-population and to be a protective factor in the non-atopic sub-population. Based on that, exposure to pets is thought to prevent asthma cases in the non-atopic population and cause additional asthma cases in the exposed atopic population Therefore the overall impact of exposure to pets is a balance between additional cases in the atopic sub-population and prevented cases in the non-atopic sub-population [15]. In this work, all factors are associated with asthma symptoms (prevalence) independent of the investigated association in the used epidemiological study.

### 2.3. Exposure Trend and Population Attributable Fractions (PAF) for Risk and Protective Factors

Observed data for age groups and 1986–2011 were used for estimation of the future trend of active smoking in Finland [17]. The same trend was applied for exposure of adults (21–99 years) to second hand tobacco smoke (SHS), whereas no trend was applied on the exposure of children (0–20 years) to SHS. The fine particle annual population weighted mean ambient concentration estimated by the European Topic Centre on Air and Climate Change (ETC/ACC) using geographical modelling was used for 2005. A trend for fine particles was estimated based on expert judgement and the recommendation by Leeuw reported in the EBoDE report [18]. For exposure to dampness and mould, as well as exposure to pets, no trend was estimated (Table 1).

In order to estimate the fraction of the asthma associated with a specific factor, the population attributable fractions (PAF) for risk factors were derived using the exposure-response estimate (RR) for each exposure. (Equation (1)) [19]. similarly, the preventive fraction (PF) for protective exposures was calculated based on PAF (Equation (2)) [20]:

(1) $$PAF=\frac{f\times \left(RR-1\right)}{f\times \left(RR-1\right)+1}$$

(2) $$PF=\frac{1}{1-PAF}$$

Baseline exposures were collected from Statistics Finland and other sources [15] and exposure-response estimates were obtained from epidemiological studies (Table 1). A detailed description of the source studies of the exposure-response function is given in the Supplementary Material (Table S1).

**Table 1:** Fraction of population exposed and risk estimates for included factors.

Scenario	Factor	Exposure in 2011	Ref.	Relative Risk (RR)	Ref.	Population Attributable Fraction (PAF) ^a^
1	Active smoking	17%	[17]	1.03	[8]	0.3%
	Second Hand Smoke (<14 y)	4%	[18]	1.32	[9]	0.6%
	Second Hand Smoke (≥21 y)	10%	[10]	1.97	[10]	4.4%
2	Fine particles (PM_2.5_)	100% ^**b**^	[19]	1.015 ^c^	[12]	11.6%
3	Dampness	15%	[21]	1.34	[11]	4.8%
4	Cat	18.5% ^**d**^	[22]	0.47	[13]	3.4%
	Cat	1.5% ^**e**^		1.67	[14]	0.3%
	Dog	22.2% ^**d**^		0.37	[13]	5.2%
	Dog	1.8% ^**e**^		2.78	[14]	0.8%

Notes: ^**a**^ in 2011; ^**b**^ 9.1 μg/m^−3^ ambient concentration; ^**c**^ risk per μg/m^3^ exposure; ^**d**^ non-atopic population; ^**e**^ atopic population.

### 2.4. Attributable Asthma Burden

The attributable burden of asthma was calculated as (i) the attributable prevalent cases and (ii) environmental burden of disease (EBD). The attributable prevalent cases (P_attr_) are the product of the prevalent cases (P) and the population attributable fraction (PAF) (Equation (3)) [19]. In case of protective exposures, the prevented cases are calculated according to Equation (3), but using PF instead of PAF [20]:

(3) $$P_{attr}=P\times PA$$

The asthma burden (EBD) was measured as undiscounted, non-age-weighted, prevalence-based Years Lived with Disability (YLD). In this work, the EBD is solely characterized by the morbidity (YLD) and the mortality is not taken into account due to the very low number of asthma caused deaths.

The background asthma YLD (YLD_Asthma_) is the product of asthma prevalence, measured as medication reimbursement entitlements in one calendar year), and the WHO asthma disability weight (DW = 0.04) (Equation (4)) [23]:

(4) $$YLD_{Asthma}=P\times D$$

EBD was calculated from the background YLD and PAF, as shown in Equation 5.

(5) EBD=P×DW×PAF=YLD ×PAF

### 2.5. Selection of Mitigation Actions

Potential mitigation actions were developed for the four identified exposures: (1) tobacco smoke, which includes active smoking and second hand smoke (SHS), (2) fine particles (PM_2.5_), (3) dampness and mould in buildings, as well as the exposure to (4) pets. All actions were modelled from 2015 onwards and the effectiveness in reducing the asthma burden was assessed as the cumulative asthma burden reduction in 25 years (2015–2040) compared to the business as usual, in which the exposure was assumed to follow the forecasted trends.

The tobacco controls included three alternative actions: (1a) a total ban of use of smoking, (1b) a 50% reduction in the tobacco consumption, and (1c) a continuous 10% annual reduction in the tobacco use. The actions (1a) and (1b) are based on Kutvonen’s work [24] and action (1c) on the Finnish Tobacco Act (No. 693/1976) (amended act entering into force on 1 October 2010) aiming at a smoke free Finland [25]. We assumed that any change in smoking is directly mirrored also in the exposure to SHS.

Since various sources contribute to the ambient fine particle (PM_2.5_) concentration and not all can be controlled easily, the selected control options focus on a specific source: residential wood combustion in areas with a population density of ≥200 inhabitants/km^2^, which is either (2a) banned completely or (2b) halved [24].

The control option for dampness and mould in buildings was defined as (3) a reduction of exposure to dampness and mould by 50 %. This action was proposed by the HealthVent study [26].

Exposure to cats and dogs presents an asthma risk to the atopic population, but a protection for the non-atopic population. To evaluate the opposing effects the exposure to pets (4) the contact with pets is increased by 50 % in the young population (<22 years), regardless of their atopy status.

Due to the non-linear exposure trends and the non-linear change in exposure in action 1c the reduction potential of all mitigation actions is presented cumulated for 25 years (2015–2040) to enable a better comparison of the total potential.

## 3. Results and Discussion

### 3.1. Historical Records of Asthma Entitlements (1986–2011) and Future Projection (to 2040)

The annual asthma entitlements have been constantly increasing from 1986 onwards. In 1986 a total of 75,213 individuals were entitled to reimbursement (prevalence 1.5%), which increased to 238,716 individuals in 2011 (prevalence 4.5%). The future projection to 2040 proposes that the number will increase to 271,424 entitlements in 2040 (prevalence 4.8%). The older population (>65 years) accounted for 24% (1986) of the total asthma entitlements increasing to 34% (2011) and it is expected to continue to increase to 37% in 2040. In contrast, the fraction of the total asthma related to the younger population (<20 years) increased from 13% (1986) to 18% (1995–2001) and decreased again to 11% (2011). It is projected that the decrease continues to 9 % in 2040.

In 2011 the number of asthma patient years attributable to exposure to tobacco (smoking and SHS) was 20,200, to exposure to fine particles (PM_2.5_) 27,703, to dampness and mould 11,584 and to exposure to cats and dogs in the atopic population 653. In the same year 5554 asthma patient years were prevented by exposure to cats and dogs in the non-atopic population. We estimated that 25% of asthma burden was attributable to the selected environmental exposures and only 2% of the total cases had been prevented by exposure to pets as a protective factor in 2011.

### 3.2. Environmental Burden of Disease (EBD) of Asthma

For comparing the significance of the asthma burden with other diseases and the mitigation actions with other environmental risk management options, the asthma entitlements were transformed also to environmental burden of disease estimates expressed as disability adjusted life years per million (DALY/million).

The total asthma burden in 2011 was 1768 DALY/million with 25% being attributable to the selected risk factors. Exposure to fine particles (205 DALY/million) was the biggest contributor to the burden, followed by tobacco (150 DALY/million (SHS 142 DALY/million and smoking 8 DALY/million)), indoor dampness and mould (86 DALY/million) and pets (cats and dogs) (5 DALY/million). The exposure of non-atopic individuals to pets prevented 41 DALY/million (Table 2). 

The total cumulative burden of asthma in Finland between 2015 and 2040 is 1874 DALY/million. Exposures to tobacco, fine particles, dampness and mould and pets caused 20%. Nearly 2% of the asthma burden is prevented due to exposure of the non-atopic population to pets (Table 2).

### 3.3. Asthma Reduction Potential 

In 25 years (2015–2040) almost 7 million asthma patient years are expected in Finland, with 1.2 million patient years being attributable to exposure to tobacco (smoking and SHS), fine particles, dampness and pets. We estimated, that the combination of the most efficient control actions (maximum reduction scenario including the actions 1.a, 2.a, 3, 4) would decrease the prevalent cases 0.7 million (60% of the total attributable cases; 10% of all asthma) (Table 2). The combination of a more feasible reduction scenario including the actions 1.c, 2.b, 3, 4 was estimated to reduce the number of entitlements by 0.5 million (43% of the attributable cases, 8% of all asthma).

**Figure 1:**
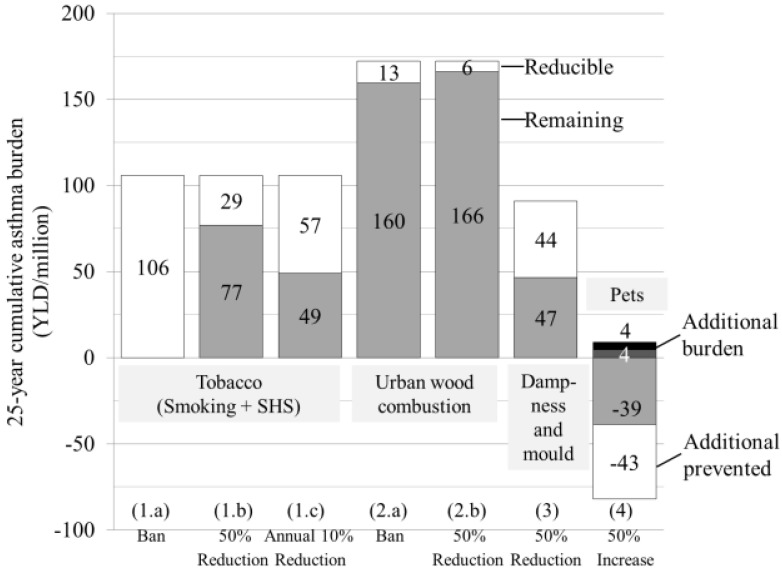
Reducible and remaining asthma burden for each target exposure and corresponding mitigation action; In case of increasing exposure to pets the attributable burden in the atopic population increases by 43 DALY/million.

The total ban of tobacco is the most efficient mitigation action (Figure 1). The annual 10% reduction of exposure to tobacco is the second efficient mitigation action reducing the 25-year cumulative asthma burden by 3%. Since the different fine particle mitigation actions tackle only the exposure due to a specific source, their overall efficiency is rather low with less than 1% reduction of asthma.

**Table 2:** Reducible and attributable patient years (2015–2040).

Scenario	Exposure change	Action	25-Year Cumulative Patient Years			
			Attributable	%	Reducible	%
	Total asthma burden		6,796,162	100		
	Attributable to risk factors studied here *		1,208,902	17.8	726,441 ^**#**^	
	**Tobacco ^a^**		383,209	5.6		
1a	Ban	100% reduction in 2015			383,209	5.6
1b	50% Reduction	50% reduction in 2015			103,642	1.5
1c	Smoke Free Finland	10% annual reduction in 2015–2040			205,930	3.0
	**Fine particle (PM_2.5_)**		624,512	9.2		
2a	Ban Wood Combustion ^**b**^	100% reduction of PM_2.5_ fraction due to residential wood combustion in 2015			45,971	0.7
2b	50 % Reduction Wood Combustion ^**b**^	50% reduction of PM_2.5_ fraction due to residential wood combustion in 2015			22,943	0.3
	**Dampness ^c^**		329,785	4.9		
3	50 % Reduction	50% reduction in 2015			160,792	2.3
	**Pets ^d^**		−128,604	−1.8		
4	50 % Increase	50% increase leading to exposure of 3.5% of atopic population and 46.5% exposure of non-atopic population			136,469	2.0
	**Feasible**	Combination of actions 1.c, 2.b, 3, 4			526,134	7.7
	**Most efficient**	Combination of actions 1.a, 2.a, 3, 4			726,441	10.7

Notes: * including tobacco, PM_2.5_, dampness and mould, pets; **^#^** including the scenarios 1.a, 2.a, 3 and 4; ^**a**^ including active smoking and second hand smoke (SHS); ^**b**^ supplementary small scale wood combustion in areas with a population density of ≥200 inhabitants/km^2^; ^**c**^ Damp and mouldy buildings; ^**d**^ including cats and dogs, aggregated for attributable cases in atopic-population and prevented cases in non-atopic population.

The pet mitigation action includes both risk (atopic sub-population) and protective (non-atopic sub-population) effects. Therefore, an increase in exposure to pets leads to additional asthma cases in the atopic population (0.2% of the total background asthma) while simultaneously decreasing asthma in the non-atopics (2% of the total background asthma).

The more feasible control scenario (combination of actions 1c, 2b, 3, 4) was capable of reducing the total burden by 8%. The reduction potential of the maximum reduction scenario (actions 1a, 2a, 3, 4) is 10% of the total burden. 

### 3.4. Burden of Asthma by Age Groups

**Figure 2:**
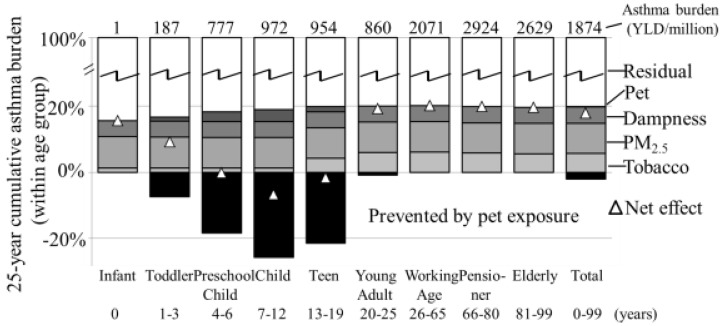
Age-specific relative 25-year cumulative asthma burden with age group total burden on top and fractions attributable to mitigation actions; the prevented burden is due to exposure to pets in the non-atopic population.

The total, as well as the attributable asthma burden is highest in the working age (26–65 years) group and lowest in the infants (<1 year) (Figure 2). The effects of exposure to pets affect only the young population (<22 years). The same is observable for exposure to pets as a risk factor. In preschool children risk and preventive effects are roughly equal. In 7–12 year old children the net prevention is 972 DALY/million, followed by teens (13–19 years; 954 DALY/million). In toddlers (1–3 years; 187 DALY/million) and young adults (20–25 year; 860 DALY/million) more asthma is caused than prevented. In the total population 1874 DALY/million of the total asthma burden is attributable to the investigated exposures. 

**Figure 3:**
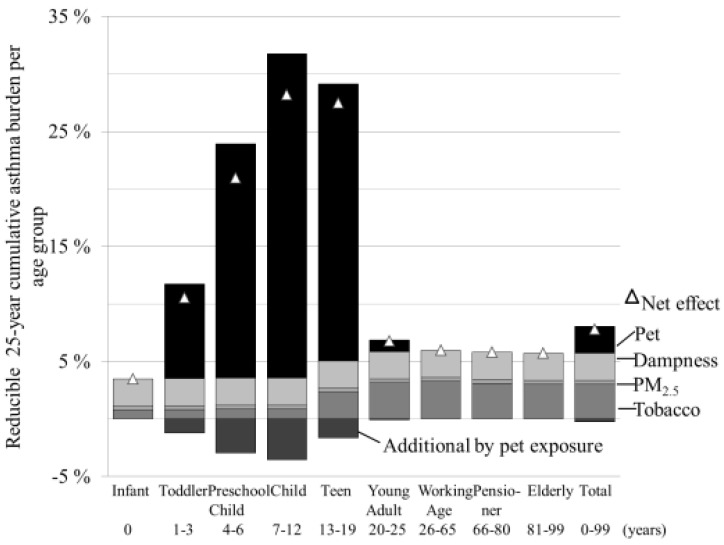
Age specific relative asthma reduction potential for the feasible reduction scenario (actions 1c, 2.b, 3, 4), with additional asthma burden caused by increased exposure to pets in the atopic population.

The effectiveness of the mitigation actions peaks for children (1–19 years), for which 10%–30% of the asthma burden could be reduced by increasing exposure to pets (Figure 3). 

If the feasible mitigation scenario (combination of actions 1c, 2b, 3, 4) was applied, the smallest reduction potential was in infants (≤1 year; 3%). The biggest fraction could be reduced in children (7–12 years; 28%). The exposure to active smoking was applied to be zero in the young age groups (<15 years), whereas the effect of exposure to pets was modelled to vanish in the older age groups (>21 years).

## 4. Discussion 

This work quantified the environmental reduction potential of asthma in Finland. While evaluation of the feasibility of the actions is critical for the implementation, it was not considered here. This work estimates merely the magnitude of the reduction potential, to be used by public health decision makers and other experts in planning. Below we discuss the sources of uncertainty in the estimates. If we accept environmental epidemiology as a reliable source of information on causal association between these exposures and asthma, the order of magnitude of the results must describe reality. Controlling environmental exposures has a clear potential for reducing asthma burden. Nevertheless, a substantial burden not attributable to these factors remains. Furthermore additional health benefits are also associated with most of the exposures, that were included in this work, and therefore the reduction potential of the mitigation actions in terms of the total burden of disease in Finland is considerably higher. 

### 4.1. Model Uncertainties

The main uncertainty in the model is the causality between the selected risk and protective factors and asthma. Although the available data on associations between exposures and asthma are increasing daily, there remain uncertainties about the causality between the associations and the relevant relative risk estimates [27]. The evidence for causality between the exposure and asthma is crucial, because if an exposure is considered, for which there is no causality, the attributable fraction of asthma and the reduction potential are overestimated. We applied the selected exposures all on the asthma prevalence estimation, even if the underlying epidemiological study assessed the relationship with asthma onset, without any scientific evidence for the accuracy of this approach. In general, the evidence for second hand tobacco smoke and fine particles (PM_2.5_) and asthma prevalence is stronger than for the other exposures. The evidence for a causal relationship between pets and asthma as a protective factor is weakest. The “German evidence-based and consented guideline on allergy prevention—updated 2014” summarizes the evidence for causality of asthma or allergy and the in this work included exposures. The guidelines concludes that exposure to tobacco smoke and motor vehicle emissions (including PM_2.5_) is associated with in increased asthma risk [28]. The evidence for causality between asthma and exposure to pets is the most controversial. Collin *et al.* report differences in risk for atopic and non-atopic asthma. In contradiction to the assumption made in this work, that exposure to pets increases the risk for atopic asthma and decreases the risk for non-atopic asthma, their study suggests a protective effect for atopic asthma and an increase in risk for non-atopic asthma [29]. Carlsen *et al.* report no association between pet ownership and asthma [30]. This controversy illustrates the difficulties in environmental burden of disease assessment and that the results of this work cannot be used in absolute terms, but give more suggestions how the rise in asthma can be controlled theoretically. The overall impact of the proposed control options in reality is going to be substantially smaller. Furthermore, the model does not account for multi-causality. All factors are assessed independently, potentially leading to an overestimation of the attributable and reducible asthma fraction. 

The complexity between exposure and outcome is difficult to characterise due to confounding factors, latency time until the onset of asthma, and uncertainties in the exposure assessment [31]. The used epidemiological studies to identify the risk estimate worked with different definitions of asthma and sources for morbidity data. The sources ranged from self-reported asthma including wheezing to health registry data. This influences the asthma prevalence and the exposure response assessment. In this study, the asthma prevalence is rather low compared to other studies due to the definition of asthma. Here asthma is defined as a KELA reviewed doctor diagnosis and strict criteria for a monetary reimbursement of medical expenses and therefore mild asthma cases are not included. The self-reported physician diagnosed asthma prevalence in Helsinki in 2007 was as high as 9.4%, which is more than double as high as in this work with 4.1% for the same year [3]. The Global Burden of Asthma study reports an asthma prevalence of 8.0% in 2001 [2]. The prevalence based on KELA data is 3.8% for 2001, indicating a substantial difference. Due to these discrepancies in the prevalence estimation, the asthma burden estimates in this work are likely to be underestimates. The definition of asthma to fulfil the KELA reimbursement criteria is very strict, but even mild asthma, which does not fulfil the KELA criteria, affects the quality of life negatively. Less conservative asthma definitions (for example based on respiratory symptoms (wheeze, chronic coughing and shortness of breath) without lung function tests or with less strict lung function test criteria will lead to substantially higher prevalence estimation. A drawback of the use of KELA data as prevalence estimation is the lack of annual re-assessment for the entitlement for reimbursement. It is not clear how the end of the entitlement is determined by KELA or in which timeframe a re-assessment of the entitlement of adults is requested. Therefore an entitlement may still be active, although the asthma symptoms may have vanished to a degree that no medication is needed anymore or that would not fall into the criteria for entitlements leading to a possible overestimation of the prevalence. Nevertheless, an increase in prevalence in the Helsinki metropolitan area has been observed in a cross-sectional cohort study from 6.5 % in 2006 to 10.0% in 2006 [32]. Again an epidemiological study suggests a much higher prevalence than the KELA data do, indicating that the later data are rather conservative. The observed increase in prevalence might be partly due to the achievements of the Finnish Asthma Programme leading to improved diagnosis and higher awareness by health care professionals and patients [32]. There is no sufficiently evident theory explaining the increase in asthma. Most often a change in the environment during programming of the immune system is discussed. Exposure to a not sufficient diversity of bacteria and other challenges for the immune system leads to a faulty programming causing allergies and inflammatory diseases [33]. Overall, it seems most likely that the increase in asthma is a result of a changing lifestyle leading to a change in exposures from prenatal development throughout the life. These exposures include fewer bacteria and animals, but more chemicals, as well as a change in nutrition. However, genetic susceptibility is likely to contribute to the increase, too.

Mortality was not included in this work due to the low number in Finland. “The Burden of Asthma” study reports a case fatality rate of 1.6 per 100,000 asthmatics in Finland, which is one of the lowest globally [2]. According to Statistics Finland the number of annual deaths caused by asthma was between 95 and 107 in 2009–2012, with a maximum of 16 deaths in the younger than 65 years olds [34]. On account of this very low number of deaths due to asthma, the contribution of mortality to the total asthma burden was assumed to be neglect able in the overall assessment.

### 4.2. Parameter Uncertainties

A crucial part of the assessment is the identification of relevant exposures, including their exposure-response relationship and exposure assessment. There are uncertainties, if the relative risk attributable for an exposure is the same for asthma onset and exacerbation is the same, additionally to the previously discussed uncertainties in the causality of association.

The exposure assessment is crucial for this work, because the exposure estimate directly influences the attributable burden. Observed data can be considered to be rather reliable, whereas modelled and estimated exposures may be less reliable. Age-grouped annual data from Statistics Finland are available for smoking in Finland enabling a reliable and accurate exposure assessment. The exposure assessment of fine particles (PM_2.5_) is based population-weighted mean ambient concentration has been used for the total population. This is not the most accurate exposure assessment, however it is deemed reliable enough for this work. For dampness and mould, as well as pets rough estimates have been used for the exposure assessment, making these data least reliable. 

Trends have been used to estimate a realistic as possible asthma forecast and exposure estimates for the whole study period. If these trends over- or underestimate the reality, this is directly reflected in the asthma burden estimates. No statistical evaluation of the trend estimates has been done. 

The observed differences in attributable, prevented and reducible asthma in the age groups are mainly due to the study design: for active smoking no exposure in the young age groups is assumed, so that there cannot be any attributable asthma in these age groups for that exposure. The exposure to pets is assumed to have effects only in individuals younger than 21 years following the available epidemiological studies. Since no effect is applied in older individuals, no asthma cases can be attributable or prevented by this exposure. 

### 4.3. Scenario Uncertainties

Here we aimed at quantifying their potential impacts to facilitate informed discussion. The work left out any considerations of the possible implementation challenges. The feasibility and the implementation of the developed actions and scenarios have not been a focus of this work and therefore the scenario uncertainties are only discussed very briefly.

The implementation of a stepwise reduction of tobacco exposure seems feasible, especially since Finnish Tobacco Act (No. 693/1976) with its amendments that entered into force on the 1 October 2010. Section 1 (20.8.2010/698) states that the “aim of the Act is the end of use of tobacco products containing compounds that are toxic to humans and create addiction” [25]. 

The mitigation of exposure to fine particles is challenging, because many sources contribute to the ambient concentration and a major reduction in the ambient concentrations needs decreases in the contribution of numerous sources. Even the change in residential wood combustion in urban areas is very challenging, because climate change policies encourage supplementary wood combustion as renewal energy source. Furthermore, wood has been traditionally used for heating in Finland and this combustion type is highly valued by the population. Therefore, reduction of residential wood combustion in urban areas would be technically readily feasible, but not easily accepted by the population.

Damp and mouldy buildings have been identified as a significant problem by the Finnish Government. In 2009, the Moisture and Mould Programme was launched aiming at reducing the moisture damaged buildings, the health effects, economic losses and to improve the methods to assess the moisture problem in buildings [35]. This encourages the idea of reducing the exposure to indoor dampness and mould. A substantial decrease in the fraction of the population being exposed to dampness and mould may be realistic as technical possibilities are known and e.g. intelligent housing technologies are improving moisture control, but only in the long term and not as quickly as estimated in this work.

One of the controversial mitigation actions is the pet mitigation action. Firstly, the evidence for an association of exposure to pets, in this case cats and dogs, and asthma is very sparse and not sufficient. This seems true for both, the presenting of a risk to atopic individuals as well as a protective effect for non-atopic individuals. Secondly, the intentional long term exposure of atopic individuals to pets, which might cause the development of asthma and/or the worsening of symptoms of asthma or of allergy in general, in order to prevent asthma in non-atopic individuals, is ethically problematic. It is unethical to take the risk of people suffering more, to prevent the suffering in another group of people. Especially considering that the evidence for exposure to pets preventing the asthma onset or symptoms is even smaller than this exposure being a risk factor for atopic people. Thirdly, it may be hard to accomplish an exposure of about half of the under 22 years olds to cats and dogs on a daily basis.

### 4.4. Validity of the Burden of Disease Approach

Although the EBD assessment is sensitive for errors and uncertainties due to the needed input data on morbidity, mortality, disease severity and exposures, errors in these data are expected to be significantly less important than the previously discussed sources of causality and parameter errors in exposure and dose-response assessment.

### 4.5. Asthma Reduction Potential and Economic Considerations

The annual reduction to tobacco smoke by 10% would decrease the total number of asthmatic patient years in Finland by 205,930 individuals cumulated for 2015 to 2040. The average cost of each asthma patient was estimated to be €1031 in 2003 [6]. Using this estimate until 2040 would make the corresponding savings, achieved by smoke free Finland action (10% annual reduction) equal to €212 million within 25 years. Obviously, the State would lose tax incomes from tobacco products, which decreases the benefit of the policy. On the other hand, tobacco exposure is not only associated with asthma, but with cardiovascular diseases and especially lung cancer, which prevalence would decrease a lot, too. It was estimated that even the total ban of tobacco products would yield an overall profit of €100 billion [24]. Therefore, it can be assumed that the gradual smoking reduction would be cost-benefit efficient, too. 

Trying to reduce asthma in Finland by controlling the ambient fine particle (PM_2.5_) concentration proves to be difficult. Nevertheless, the 46,000 patient years reduced by a total ban of residential wood combustion in urban areas would reduce the medical costs for asthma in 25 years by more than €73 million. The net profit of this policy would be between €0.5 and €1 billion [24]. Fine particles are associated with not only asthma, but similarly as tobacco smoke also with cardiovascular and other diseases. The benefits due to a reduction in these outcomes are significant. 

The net profit of the dampness and mould mitigation action is going to be smaller, because the remediation of dampness damaged buildings requires investments. Nevertheless, in about 160,000 asthmatics symptoms would be prevented with this policy, which would result in about €165 million savings in medical expenses for asthma medication within 25 years.

The monetary impact of the pet mitigation action is less straight forward. On the one hand, about €6.5 million for medical reimbursement would be saved due to the decrease of asthma patient years, but on the other hand the costs due to asthmatic and allergic symptoms of the atopic individuals would rise with alone 0.7 million € spent on the additional asthma patient years. Additionally money would be needed for campaigns promoting the idea of increased exposure to pets in day-care centres and schools. Additionally costs for the pets are not clear and, if it is the private owner, the school or city or if there would be a governmental programme paying actually owning and taking care of the pets.

## 5. Conclusions

The prevalence of asthma in Finland rose from 1.5% in 1986 to 4.5% in 2012 and a further increase to 4.8% (2040) is projected. In 2011 about 25% of all asthma was attributable to the studied risk factors: tobacco (SHS 142 DALY/million; smoke 8 DALY/million) fine particle (205 DALY/million), indoor dampness and mould (86 DALY/million) and pets (5 DALY/million). This study quantified the reducible fraction of asthma in Finland by adjusting of exposures to the studies factors. In addition, pets were included also as a protective factor. The results indicate that up to 10 % of asthma burden can be reduced within 25 years by combining the ban of exposure to tobacco and residential wood combustion in urban areas, halving of exposure to dampness and mould, and doubling of exposure to pets (most efficient scenario). Especially the ban of tobacco was shown to be effective in reducing asthma with a reduction by 6 % (5.3 % SHS and 0.4 % smoking). The mitigation actions developed for fine particles (PM_2.5_) are least effective with a reduction of less than 1 % of the total asthma burden within 25 years, because each option targets only one specific fine particles source and therefore reduces fine particles exposure only slightly. The results suggest that not only a better self-management of asthma and better medication, which were the aims of the Finnish asthma programme, should be considered as ways to tackle the increasing asthma problem, but also controlling exposures associated with asthma. Better understanding of the disease aetiology of asthma would enable a differentiation between exposures being associated with either onset or exacerbation, or both.

## Supplementary Files

Supplementary File 1
