# Supplementary material for: Environmental Asthma Reduction Potential Estimates for Selected Mitigation Actions in Finland Using a Life Table Approach
Source: Int J Environ Res Public Health. 2015 Jun 9;12(6):6506–22. doi: 10.3390/ijerph120606506 (PMC4483713; doi:10.3390/ijerph120606506)
Supplement: Supplementary File 1 [file ijerph-12-06506-s001.pdf]

Environmental Asthma Reduction Potential Estimates for Selected Mitigation Actions in Finland Using a Life Table Approach

Table S1. Summary of the epidemiological studies on primary exposures selected as asthma mitigation targets.

Stressor	Association	Response	Definition of Asthma	Age (Years)	Exposure Unit	ERF Parameter	ERF (CI)	Country	Study Design and Size	Author, year
Second Hand Tobacco Smoke	Risk	Onset	self-reported and physician diagnosed	<14	yes/no	OR	1.32 (1.24–1.41)	worldwide	Meta-Analysis of 29 studies	Cal-EPA, 2005
Second Hand Tobacco Smoke	Risk	Onset	physician diagnosed and/or KELA reimbursement	≥21	yes/no	RR	1.97 (1.19–3.25)	Finland	Case-Control study (521 cases, 932 controls)	Jaakkola *et al.*, 2003
Active Smoking	Risk	Exacerbation	self-reported asthma	13–14	yes/no	OR	1.2 (0.7–2.1)	France	Cohort study (15171 subjects)	Annesi-Maesano *et al.*, 2004
Active Smoking	Risk	Exacerbation	self-reported asthma	≥20	yes/no	OR	1.03 (1.03–1.04)	Norway	Cohort study (65717 subjects)	Langhammer *et al.*, 2000
Particulate Matter ($PM_{2.5}$)	Risk	Onset and wheezing	physician-diagnosed	all ages	10 μg m^{-3}	RR	1.16 (0.98–1.37)	worldwide	Meta-Analysis of 5 studies	Anderson *et al.*, 2013
Dampness and Mould	Risk	Onset	physician diagnosed and first-time diagnosis	all ages	yes/no	OR	1.34 (0.86–2.10)	worldwide	Meta-Analysis of 4 studies	Fisk, Lei-Gomez and Mendell, 2007

Cat	Protection	Onset	self-reported by parents	7–16	yes/no	OR	0.47 (0.14–1.58)	Finland	population-based cross sectional study (1,400 subjects)	Hugg *et al.*, 2008

Table S1. *Cont.*

**Stressor**	**Association**	**Response**	**Definition of Asthma**	**Age (Years)**	**Exposure Unit**	**ERF Parameter**	**ERF (CI)**	**Country**	**Study Design and Size**	**Author, year**
Dog	Protection	Onset	self-reported by parents	7–16	yes/no	OR	0.37 (0.13–1.1)	Finland	population-based cross sectional study (1 400 subjects)	Hugg *et al.*, 2008
Cat Allergy	Risk	Exacerbation	self-reported symptoms	7–8	yes/no	OR	1.67 (0.83–3.37)	USA	Case-Control study (128 cases, 111 controls)	Olmedo *et al.*, 2011
Dog Allergy	Risk	Exacerbation	self-reported symptoms	7–8	yes/no	OR	2.78 (1.29–5.99)	USA	Case-Control study (128 cases, 111 controls)	Olmedo *et al.*, 2011

© 2015 by the authors; licensee MDPI, Basel, Switzerland. This article is an open access article distributed under the terms and conditions of the Creative Commons Attribution license (http://creativecommons.org/licenses/by/4.0/).
